# The Hospital Nursing Supplement

**Published:** 1891-04-04

**Authors:** 


"The Hospital" Nursing Mirror.

Being the Extra Nursing Supplement of "The Hospital" Newspaper.

Contributions for this Supplement should be addressed to the Editor, The Hospital, 140, Strand, London, W.C., and should have the word "Nursing" plainly written in left-hand top corner of the envelope.

### En Passant.

Gloucester District Nurses.—The Very Rev. the Dean of Gloucester presided at the annual meeting of this society, when the Rev. A. C. Eyre read the report. Persons nursed during last year numbered 255; the expenditure was £251. The society has lately obtained the services of Miss C. G. Evans as Superintendent, and she is proving herself a good organiser.

Sheffield Infirmary.—An excellent scheme of training nurses was inaugurated at Sheffield about a year ago; it had only one fault; but unfortunately that fault was a serious one, and threatens to mar the whole scheme. There was no Lady Superintendent appointed. For some time the head nurse, Miss Tomlinson, has been doing her best without the title or the authority to superintend the nurses' work and the state of the wards, and some of the guardians thought to put the wrong right by giving Miss Tomlinson the station and title she needed. The motion was put, and there were 9 for and 9 against, and the Chairman gave his casting vote against. Better luck next time, nurses!

Rural Nursing Association.—An influentially-attended meeting was held at the Kenton School-Room lately for the purpose of taking into consideration the advisability of forming a branch of the Rural Nursing Association for the district. The chair was occupied by the Rev. the Right Hon. Earl of Devon, and there were also present the Countess of Devon, Major and the Misses Courtenay, the Rev. W. S. P. Bingham (Rector of Kenton), the Rev. E. C. Bond, and others. Mr. Broad-Bissell moved, "That as an experiment a branch of the Rural Nursing Association be formed for the parishes of Starcross, Kenton, and Powderham for one year. Mr. Waterfield asked whether it would not be well to include Cofton in the district. Mr. Bingham thought it would be desirable, and Mr. Broad-Bissell accepted the addition to his resolution, which was then unanimously carried. A subscription list was commenced, and £27 collected in the room. We are glad to see the R.N.A. developing so widely and so well. Lady Victoria Lambton has lately been writing and speaking in favour of a new branch of the Association for St. Clears.

Cruelty.—"It was positive cruelty to behave like that in a house where anyone lay dying." "She has quite destroyed my belief in nurses." "It was brutal behaviour." So the conversation went on round a luncheon table, the subject being the conduct of a private nurse working in London on her own account. This nurse was called up late in the night to go to a case where one nurse was already in attendance. The household was greatly upset, the house was small, but the extra nurse would make no allowances; she bullied the poor little maid-o'-all work, she demanded her meals at awkward times, in fact, she behaved most cruelly, though doubtless she would be astonished to hear such an adjective applied to her conduct. We have inquired into the facts of the case, and have put the name of the nurse on our black list. What we would point out as a warning is that there is nothing against her in her nursing duties, though the doctor who attended the case will never call her again, for she frightened the patient, increased the awful grief of the distracted family, and has in the eyes of a large circle of friends discredited her profession and her comrades. Such are the results of the total absence of womanly sympathy, and concession, and toleration; in a nurse they amount to cruelty.

Bolton Nursing Association.—The Mayor presided at the annual meeting of the Bolton District Nursing Association, held in the Town Hall on March 19th. Mr. W. W. B. Hulton read the annual report of the Association, which is undoubtedly progressing in a most satisfactory manner. The total number of visits made by the nurses during the year was 11,558, an increase of 8,796 on the previous twelve months. The number of cases attended was 532, as against 303 in the previous year. No fewer than 319 of these cases had been sent in by doctors. During the past year the Association had been affiliated to the Queen Victoria Jubilee Institute for Nurses, which placed them in a better position than would otherwise be the case. The balance-sheet submitted was most satisfactory.

Hospital for Women, Soho Square.—The Bishop of London, on last Saturday afternoon, March 28th, at the request of the chaplain, the Rev. Charles Parkhurst Baxter, held a special confirmation at the Hospital, in the Shaftesbury Ward, when five of the patients received the apostolic rite of laying on of hands. His Lordship delivered a most impressive address to the candidates and to the patients present who could be removed with safety from their respective wards. All present joined heartily in the service. The patients have expressed their gratitude to his Lordship for coming among them and ministering to their great need in their sickness. The Rev. Canon Ingwell, a member of the Committee, was also present.

A Disgrace to England.—The Local Government Board inspection into the state of the Steyning Workhouse reveals a scandalous state of things, which is peculiarly disgraceful because it is the Union Infirmary of the rich town of Hove, known better as West Brighton. The premises and nurses were dirty and untidy, and the patients infested with vermin. One paralysed patient had only been taken out of bed once in nine months; his back was raw, so were his armpits. Sarah Privett, aged 60, said she had been nineteen years in the infirmary, when she resigned; she had no one to help her but the paupers. Miss Von der Goltz said she came to the infirmary as nurse in January, and found it in a shameful state; the beds were dirty, the lavatories filthy, and vermin swarmed. We should like to know who is the medical officer, and who the guardians of this Union? Are they all blind, or have they some worse affliction?

Short Items.—The Nurses' Club, 12, Buckingham Street, Strand, has secured a second room, as it had outgrown its present limited space.—The *Daily Telegraph* of March 25th had a very strong leader on the insanitary condition of St. Bartholomew's Hospital.—A lady writes to the *Times*, signing herself "One Who has Suffered," suggesting that trained nurses ought to be forbidden to tell dreadful stories to their patients about former patients. To tell horrid stories is a most injurious custom, and much too common.—Dr. Downes will in July open a discussion at Newcastle on Workhouse Infirmary nursing.—The Matron of Guildford Hospital who sent infected clothing by parcel post had to pay the costs of the action brought against her.—Mrs. Humphreys-Roberts has been elected Superintendent of the new Nursing Association at Denbigh.—The Countess of Zetland last week visited St. Patrick's Nurses' Home, Dublin, and was shown round by Miss Watson.—At the Spring Cork Assizes a true bill for manslaughter has been found against Catherine Horgan, who, last August, administered an overdose of paraldehyde to a patient.—Miss Woodruffe has started a Nursing Institute in the South Mall, Cork.

### Lectures on Surgical Ward Work and Nursing.

By Alexander Miles, M.B. (Edin.), C.M., F.R.C.S.E.

Lecture XIX.—SPECIAL OPERATIONS.

Causes of Rise of Temperature after Operations.—One of the reasons I gave for dressing a wound after operation was "an unexplained rise of temperature." This leads me to speak of the more common causes of elevation of temperature within the first few days of operation. 1. We are never alarmed if a patient's temperature goes up a degree or two on the evening of operation, as experience shows that such a rise is very common, without being an indication of any serious condition, and that the temperature soon falls again. 2. There is no commoner cause of a sudden rise of temperature in surgical patients than constipation, and you should always inquire into the state of the bowels before going further afield for an explanation. Should you have reason to suspect that this is the cause, a free purge of castor oil or Henry's solution, supplemented by a large enema of soap and water, will often settle the point. If the free evacuation does not bring down the temperature some other explanation must be found. 3. Tension is another very common cause of elevation of temperature. A tight stitch, a blocked drainage tube, bagging of pus, the caking of discharge in a deep dressing, or any other condition preventing the free escape of discharge, very soon manifests itself by raising the temperature. Obviously the best way to treat such a case is to dress the wound and remove the obstruction. 4. Related probably to tension is the rise of temperature which one sometimes observes in cases where cavities have been stuffed with iodoform gauze or other stuffing, and in which the removal of the stuffing is usually followed by a fall in the temperature. 5. The onset of sepsis, or of any of the septic diseases, such as erysipelas, septicæmia, or pyæmia, is usually ushered in by a sudden rise of temperature among other things, and it is this cause you most dread and guard against in surgical practice. 6. The high temperature may be due to some condition quite apart from the patient's surgical affection, such as a chill, the onset of a bronchitis or pneumonia, or some such medical affection. The history and general condition of the patient, taken together with the physical examination of the chest or other region indicated, will be sufficient to decide the point. Allied to this set of cases is the rise and fall of temperature so common in tubercular patients, whether the surgical affection be of that nature or not. 7. Excitement of any kind is a not uncommon cause of elevated temperature in neurotic patients. For example, the receipt of bad news, the visit of a friend, the admission to the ward of a serious accident, or a death in the ward may temporarily raise the temperature in some patients.

Nursing of Special Cases after Operation.—In certain serious operations much of the success depends on the after treatment, and especially on that part of it which devolves on the nurse. In some of these special training is necessary, and in all of them experience in nursing ordinary cases is absolutely essential in the nurse who is sent to take care of the subjects of them. An elementary knowledge of the pathology of the affections which have rendered the operations necessary, assists the nurse greatly in intelligently performing her duties.

1. Hernia.—(*a*) Strangulated hernia: In the condition of hernia or rupture, a small portion of the bowel has escaped from the abdominal cavity through some weak point in the walls. The commonest localities in which such projections take place are the groin and the navel. The canals or openings through which these knuckles of bowel come are usually bounded by firm fibrous bands, and the gut is in constant danger of being nipped between these, giving rise to the condition of strangulated hernia. When this occurs the onward passage of the fæces is, of course, arrested. In addition, the blood supply to the portion of bowel outside the constricting band is cut off, and this produces gangrene, or death of it. The inevitable result of this is that the contents of the alimentary track escape into the abdominal cavity, giving rise to septic inflammation of the peritoneum or membrane lining that cavity. In strangulated hernia the bowel has been aptly compared to a piece of wet blotting paper, and this being so, you will readily understand that the patient's life is in imminent danger. If you bear in mind this fact with regard to the state of the gut in your patients who have been operated on for this condition, it will enable you to appreciate the importance of attending to some of the directions for the treatment given you by the surgeon. The main indication in the treatment is to secure for this devitalised portion of intestine absolute rest. Now, as the work of the bowel is to absorb the digested food, and pass on the unabsorbable remains, the surest way of keeping it at rest is to starve the patient, so that there will be nothing either to absorb or pass on. The bowel may be still further quieted by the administration of opium, either hypodermically or by the mouth; but this, of course, you will leave to the doctor. For the first day or two after the operation, the patient must have nothing by the mouth save sips of water or small pieces of ice to suck, then a small quantity of milk may be given, and later still, some light pudding and beef tea. Avoid anything solid for at least a week, and above all don't give any laxative medicine whatever. If the patient be well at the end of eight or nine days an enema, followed by a moderate dose of castor oil, may be given if the bowels have not already acted spontaneously.

(*b*) *Radical Cure of Hernia.*—The treatment of a patient who has undergone this operation differs from that of which we have just considered only in the duration of the period of starvation. This depends on the fact that here the bowel itself is uninjured, the operation consisting merely in closing up the opening through which the hernia protrudes, and so preventing the patient ever having a strangulated hernia. The bowel must be kept at rest for a time to avoid the necessity for straining, which might burst open the wound in the skin and abdominal walls; but this, although astringent at first, need not be so prolonged as in the more grave condition just described.

### Princess Christian's Daughter.

Miss E. Durham, Farringford, Freshwater, Isle of Wight, acknowledges the following additional subscriptions towards a wedding present for Princess Louise of Schleswig-Holstein to be given as a proof of the gratitude of nurses for the interest Princess Christian has ever taken in their progress. From March 23rd to 30th the following sums were received:—

Superintendent Georgina Kinninmont, 5s.; Superintendent Agnes W. Brittain, 5s.; Emma Dormer Pryhe, 5s.; Miss Daniell, 2s. 6d.; Matron Emily Angove, 5s.; Maria Woodward, 5s.; Matron E. A. C., 2s. 6d.; Sister Tempe N. Roche, 2s. 6d.; Sister F. H. R., 1s.; Annie McComere, 2s.; Nurse Lucas, 2s.; Nurses (1s. each): Staff Nurse M. Bendall, L. Cox, Bouty, Willson, Bingham, Foster, Evans, Collins, J. M. Davis, C. P., M. B., Lucy, May, J. C. Child, Julia Whiles, M. Dix, Amelia Waters, A. L. Moore, M. E. Hooper Smith, Ruth Ann Phillips, S. Watts, S. Graham, F. Storm, A. N. Furguson, C. R. Attfield, Cordelia Duffield and nurses, Wilson-Whitmore; Charles and Gregson send 11s.

[Our readers are warned that next week will be the last time subscriptions will be acknowledged in these pages.]

### Presentation.

Dr. Humphreys, the Medical Superintendent of the Fever Hospital, Bradford, is leaving to take up a private practice at Cheltenham. His departure is regretted at the hospital, especially by the nursing staff, who, on Monday, presented him with an ivory inkstand and a hall gong, with an inscription significant of their "respect and gratitude," and accompanied by a letter signed by each individually in which the same sentiments were set forth more fully.

### The Eastern Hospital Scandal.

Part of the evidence for the defence was heard last week.

Miss Susan Farnham was the first witness called for the defence. She said: I am a nurse at the Eastern Hospital, and have been there three years and five months. I was promoted to charge nurse in May last year. I have been assistant nurse in every ward except Gladness and Hope Wards. On May 1st, 1890, I opened Truth Ward as assistant nurse, and in the course of that month I became charge nurse. As to the food generally, I did not consider it at all bad. During 1889 the bread was very good. The nurses have the same food as the patients, but not the same butter. On one or two occasions the bread was rather dark, but I had no fault to find with the taste. The patients had boiled mutton, but the nurses have roast mutton sometimes and sometimes other kinds of meat. The bread is never served new. The bread in 1890 was also good. I never tasted sour or mouldy bread while I have been at the hospital. I do not think I ever saw it mouse-eaten. It was customary to bathe four children in one bath, if they were free from sores or discharges from the nose or ears. So far as I know, no patients suffering from different diseases were ever put in the same bath. The poultices were made in broken tea-mugs and porringers. Tooth brushes were used for cleaning the children's teeth and also for adults, if they ask for them.

Cross-examined by Mr. Eldridge, the witness said she was originally a housemaid at Charing Cross Hospital, and afterwards went to the Eastern Hospital as an assistant nurse. Her sister, who was also at the same institution as an assistant nurse, had had no previous experience of nursing. The bread supplied was never served new; it was usually two days old.—Do you mean to say it was never a week old? No, it was not. It was not fancy bread, but good, wholesome bread.—Baked specially for the hospital? I suppose so. The milk used to be brought into the corridor just outside the ward. The linen basket was lined with zinc, and was movable. It was always kept under the window in the short corridor. In the summer months the milk came up scalded, and in those cases I left the can open, and did not put the lid on. The windows in the short corridor were always open day and night. I put the milk into the refrigerator. With thirty patients I expected to get fifteen pints in the morning and fifteen pints in the evening. I always had enough. I never was short of milk. Some of the elder patients put water with their milk.—Now "sick" and "middle" diets on the dietary table allow two pints per patient? I always had sufficient.—That is not an answer. Did you ever get the milk mentioned in the dietary table? I never had thirty patients.—What was your greatest number? I believe twenty-six.—Did you, then, have twenty-six pints in the morning and twenty-six in the evening? I suppose not.—Mr. Hedley: You never got twenty-six pints at a time? Your can only held twenty pints? Yes.

Questioned as to her past, witness admitted that the child Albert Farnham was hers. She was not a married woman. The witness was severely cross-examined as to the alleged indecent remarks made to her by Simpkin. She remembered a dance taking place at Christmas after a children's Christmas tree, some of the patients being in bed. Dr. Collie was present for some time while the dancing was going on. The Matron, who had been present earlier, was not present at the dancing. She did not know if it was true that the Matron forbade the dancing, or that the nurses went in a body to Dr. Collie and got his permission.

Miss Sapphira Spraughton (whose unfortunate name caused a smile when announced) was the next witness. She went to the hospital as a ward maid in 1875, but had acted as a nurse for the last nine years. She did not remember Nurse Harrington saying the beef tea jelly was bad, neither did witness say she supposed they had so much in store that they wanted to get rid of it. She never said to nurse Gilman that it was too much trouble for Dr. Collie to attend to the children's abscesses, and that when she spoke to him about them he left the ward in a furious temper.

Cross-examined by Mr. Eldridge: There had been a new tin supplied to the ward lately. The dirty linen basket was kept in the corridor between two open windows. She had two milk cans, one of which held thirty-six pints and the other twenty-four pints. The nurses had a pint measure to measure the milk. She had known the milk to go bad on one or two occasions. She never remembered any one coming in from another ward to her to borrow food or clothing. Altogether, as far as she knew, everything was as good as it could be. Mr. Eldridge: Almost the place to go to for a holiday. (Laughter.)

Ellen Long said she had been four years in the Eastern Hospital, and before that was three years in the London Fever Infirmary as nurse. Speaking generally, the quality of the food supplied to the wards was very good, and no fault was to be found with the bread, milk, meat, and potatoes. She recollected, however, that on one occasion in August of last year she found the beef tea not to be very good, and she ordered some beef tea jelly instead. Witness had known instances where the diets had been short; but that was remedied by an application for further food being at once made to the stores and kitchen. She had occasionally known bad eggs to be sent up, but they were always changed. All the patients were well and comfortably clothed, and she had never known any complaints on that score. The bathing arrangements were carried out with the greatest care, and she had never heard of four children being bathed together. Patients had asked to have water mixed with the milk. The inquiry was again adjourned.

### Examination Questions.

Some thirty-eight answers were received to last month's question, and the prize has been awarded to Nurse Elizabeth Braybrooke, of Lambeth Infirmary, to whom we have sent Robinson's "Lectures to Nurses." Amongst those deserving honourable mention are Nurse Huxham, Nurse E. M. Leake, "Nurse Frances," Nurse Ethel Wilson, Nurse Rose Carter, Nurse Anna Elizabeth Day, Sister Hart, and Nurse Mary Silvester. And here we must admit that many admirable answers were disqualified because they commenced with a dissertation on the causes and symptoms of croup, whereas the question was simply "If a child is seized with croup, what should be done while waiting for a doctor?" Then some of the answers did not take the remedies in the proper order; according to Dr. J. W. Martin they stand thus: (1) An emetic; (2) a bath; (3) poultices or fomentation; (4) warm moist atmosphere. Some of the answers started with the steam kettle and finished up with the ipecacuanha. The question for next month is:—"How must the disinfection of a room be carried out after the convalescence of a scarlet fever patient?" Answers must reach this office (140, Strand) by April 18th; they must be short and written on one side of the paper only; the full name and address of the writer must accompany each paper.

Prize Answer.—If possible make the child vomit by giving one or two drachms of ipecacuanha wine in warm water sweetened. Hot drinks should be given until vomiting occurs. Should there be no ipecacuanha wine at hand, a little salt and water or mustard and water may be given. The child's feet should be put into hot water and mustard, or better still, the child should be put sitting in hot water, the shoulders being covered with flannel. Then a poultice of three parts linseed and one of mustard should be applied to upper part of chest and throat. The room should be kept warm and the air moist by placing a kettle on the fire and the steam being well conducted into the room by a tube. A roll of paper will do well for a tube for a time.

### American News.

New Jersey.

Dear Mr. Editor,—You are so interested in our sister nurses on this side of the Atlantic that I must give you a description of a "graduating class" which I had the pleasure of attending a few nights ago.

It seems that after the nurses of the City Hospital here have undergone a two years' course of training and passed certain examinations they not only receive a diploma, but also a very pretty gold medal. On this occasion the presentation took place in a public hall, which, of course, was crowded. The stage was very prettily decorated with palms and flowers, and the hall adorned with flags.

Punctually at eight p.m. the Matron, clad in a simple but exceedingly pretty grey gown and large muslin mob-cap, made her appearance, closely followed by the "graduating class," which consisted of twelve uniformed nurses. With said uniform I fell violently in love; it consisted of dark blue prints, little muslin Normandy caps, and snow white kerchiefs pinned across the bosom (as was the fashion in the days of our grandmothers), large white regular nursing aprons, minus the bibs, and white sleeves buttoned above the elbow. You may be sure I regarded their costume with the greatest interest. Each had pinned at her waist, in honour, I suppose, of the festive occasion, a few loose roses. As they sat there awaiting their honours (for which I doubt not they had worked hard), looking so neat, sweet, and simple, I felt proud to think that I, too, belonged to the nursing profession, and although those twelve women were strangers to me, and of different nationality, yet there was a strong bond between us.

After the band had played an overture, one of the Newark clergymen offered prayer. The opening address was then made, the details of which, however, I will not weary you with. Referring to the gold medals which were presented to the nurses by the Board of Trustees of the Hospital, the speaker said: "To-night a badge is given to each of the graduating class, which she may wear, and by which her title to confidence and employment may be known. A cross, emblematic of self-sacrifice, upon it a dove flying, as did the one which Noah loosed from the Ark, when it returned bearing in its bill a budding branch. The dove is an emblem of tenderness and affection, the budding branch an emblem of hope—the story told in the motto inscribed 'Affero Spero,' 'I bring you hope.' The language of the whole is, 'With self-sacrifice following Him who died upon the Cross, and seeking to be like the dove in tenderness and sympathy.' Join with me, friends, in the prayer that everyone who bears this badge may illustrate all which its mute language conveys."

The report of the training school was next made by the Matron, Miss Dunlop. It opened in the year 1887, each succeeding year showing a record of increased numbers, funds, etc., which must have been very encouraging to her and all interested.

Then followed a well-written essay on the hard task of nursing the sick poor in their own homes, which was read by one of the graduates. The clergyman already referred to next delivered an address. He spoke of the services of Florence Nightingale and her band of 93 trained nurses during the Crimean war, and of the impetus this had given to trained nursing. He drew upon our risible faculties by his description of the never-to-be-forgotten and famous "Sairey Gamp," and alluded to the great advances made in nursing in the disappearance of such (to put it mildly) *thirsty* ladies and of their replacement by the educated, skilled trained nurse who is a lady and a Christian; the nurse of to-day, he said, had done much to rob the sick-room of its terror. He closed by paying a touching tribute to the services of the trained nurse, and then wished the graduates all success and prosperity in the work which they had chosen.

The diplomas and badges were then presented by the Mayor of the City, the Matron promptly fastening on each medal as received by the different nurses.

In the eyes of a true American any occasion such as this would not be considered complete without flowers, so each nurse had the joy of receiving at least one basket or bunch of exquisite roses, sent by friends and admirers, a very graceful attention, I think—do you not agree with me? The ceremony closed as it had commenced, with music. Now I must bring this long letter to a close, or you will be wearied of it.

You ask if I have been to any of the large New York hospitals yet? I am ashamed to answer "No." Soon I hope to do so, however, and you may be sure I shall give you a full description of all I see.

The eight hours working-day has been applied to nurses in two hospitals in this country; it is attained by the nurses working in three shifts. In the Memorial Hospital at Orange the three watches are six a.m. to two p.m., two p.m. to ten p.m., and ten p.m. to six a.m. The other hospital is at Detroit, and in both the eight-hours system has so far been successful. At Detroit the nurses are not paid, and they have to do private nursing during their period of probation, so that the hospital gains by their services, and can afford to keep a very large staff. More news in next letter.

Bessie.

### Everybody's Opinion.

[*Correspondence on all subjects is invited, but we cannot in any way be responsible for the opinions expressed by our correspondents. No communications can be entertained if the name and address of the correspondent is not given, or unless one side of the paper only be written on.*]

### PENSIONS FOR SERVANTS.

The Secretary of the General Domestic Servants' Benevolent Institution, 32, Sackville Street, Piccadilly, writes: "In reply to 'District Superintendent,' of March 7th, allow me to call attention to this Institution, which was established in the year 1846 for the three following purposes, viz.: 1. To grant pensions to domestic servants when past work. 2. To assist domestic servants when out of situations through no fault of their own by small grants; to reinstate them in service, and to assist others in urgent cases of temporary distress. 3. The opening of a registry where masters, mistresses, and domestic servants can make their wants known, and through this medium provide themselves with good servants and servants with situations. Since the formation of the institution 288 pensioners have been elected, 98 of whom are now in receipt of £15 to £20 each per annum. Upwards of £37,500 has been distributed in pensions and temporary relief to aged, infirm, and distressed domestic servants, and has a reserve fund of £22,090. The amount of subscription varies according to age at time of entry—from 3s. to 8s. per annum for females, and 5s. to 10s. per annum for males, thus placing this Society within the means of all classes of domestic servants. Members are received from all parts of the United Kingdom. I shall be pleased to forward papers to any of your readers who may wish for further information on this subject on their making application for same."

### GRUMBLING PROBATIONERS.

"A Matron" writes: "I enclose a letter from a probationer cut from a contemporary. To my mind, it is very sad and humiliating to see how the spirit of grumbling and discontent is rife amongst nurses in the present day, and how almost entirely the self-sacrificing spirit which used to animate the better class of nurses is dying out. I cannot help saying that I think far too much prominence is given to nurses and their complaints. In every hospital there will be at some time or another an ill-conditioned nurse or probationer who will, like an ill-conditioned patient in a ward, affect, more or less, all her fellow-workers. She then proceeds to publish her grievances in the 'papers,' and causes more trouble and annoyance to the hospital authorities who have, unhappily for themselves, engaged her on their staff, than can easily be set right. I should advise these grumblers to read the 'Life of Agnes Jones,' who was the first lady nurse trained at St. Thomas's Hospital many years ago. Let them see the real hardships that young and wealthy lady endured without a murmur or complaint, and then I think they will feel ashamed of their selfishness and discontent. This probationer, whose friends lay such a stress upon the fact of her being a 'lady,' does not show much of that spirit of honour and loyalty which one naturally expects from a gentleman or gentlewoman. Her only ground of complaint seems to be that in an emergency she was asked by the Matron to do a little extra work. I remember during my year of training being asked by my Matron to go to the fever wards and take night duty after having had a hard day's work in an accident ward. I was tired, I admit, but only too pleased to be able to help the Matron in a difficulty, neither did I find my 13 hours at a stretch to be the unbearable punishment which this poor Plymouth pro. seems to have felt hers. Certainly whatever hardships we had to endure (and there are hardships in every hospital) it never occurred to us to write to the papers grumbling and complaining and bringing discredit and contempt on our profession, and trouble and annoyance on those who are entitled to our loyal help. I may add that I know nothing whatever of the Plymouth Hospital nor of its officials, but I have always heard the Matron spoken of by those who have worked under her with much affection. I enclose my card."

---

### A NURSE'S HOURS.

"E. A. S." writes: May I make a few remarks on the letter of "One in the Ranks?" I do not agree with those who are for ever "grumbling," but if nurses would all pull together. this vexed question of long hours would settle itself. I have before remarked that the greatest number of women do not take up nursing from a spirit of devotion; not a bit of it, they take it up because they want bread, because they are tired of home, because of some trouble, real or fancied, and, don't be shocked, but it is quite true, love affairs drive many into hospital work, and a love affair will make the greatest number of nurses forget their devotion to the "blessed patients," be those devoted nurses young or old. Fifteen hours year in and year out per day are far too many, and too much should not be demanded. I know full well how matrons are blamed for "overworking" their nurses; how can they help long hours, when for getting an extra probationer they are sat upon by the committee and bullied over a few extra shillings, though the said committee spend the funds in a very often lavish manner; for instance, fine white-handled knives and silvered forks for patients?—quite true. Nurses should have no more than twelve hours a-day, and that is four more than the lords of creation care to work; every day out of those twelve hours nurse should have some time to herself, as can be best managed. The very fact of the impure air, the constant standing, is enough to tire any woman, and to many of our young probationers—who never did a day's work before they came to the hospital—would not break down if they had a fair chance given them.

"ONE ON THE ACTIVE LIST" writes: If many such ultra-humble-minded individuals existed as the lady who signs herself "One in the Ranks," the efforts of those interested in the nursing world, to raise the status of nurses generally, would be somewhat unappreciated. While heartily concurring with her that the Divine example is the best and only guide out of the many difficulties that beset a nurse's path, I cannot but remember the old saying that "God helps those who help themselves." Therefore, let them use the means He puts in their hands for their happiness and prosperity in this life, which, though at best a short one, ought to be capable of yielding a large amount of joy and gladness to themselves as well as their neighbours, doubly so if His gracious gifts of health and strength are used and not abused in that state of life unto which He has called them. If "One in the Ranks" should ever have charge of a hospital, I must say I should be very sorry for her nurses if she adhered to her ideas of what they ought and ought not to do with regard to length of working hours. I should also pity the patients who were nursed by such jaded and weary attendants as they perforce would be. All honour to the promoters of schemes of help for nurses who, till lately, had few to fight their battles, and who—or at least a large majority of them—appear extremely grateful to their champions.

### Notes and Queries.

TO CORRESPONDENTS.—1. Questions may be written on post-cards. 2. Advertisements in disguise are inadmissible. 3. In answering a query please quote the number. 4. If a private answer is desired, a stamped addressed envelope must be forwarded.

NOTICE.—We must really ask our correspondents to be more particular in enclosing name and address. Constantly we receive queries or letters with no name attached.

---

### Queries.

(46)—Can any one recommend two unfurnished rooms and kitchen, with clean, respectable people? Wanted by two private nurses. Rent must be moderate.—*Miss H.*

(47)—An old woman has a son, 14, who is subject of talipes with some paresis of legs, causing awkward gait. Is there anywhere an institution into which he could go in order to learn a trade of any sort (such as basket-making or sitting work)? He will never be fit to fight his way in the world with others for ordinary clerks' posts. Fair scholar. Seventh standard boy.

### Answers.

(38)—I have had rather more than six years' experience with very young babies, but have never found that they could go from eleven p.m. to seven a.m. without food. They require feeding every four hours.—*C. J. Forrest.*

(38) *Feeding Babies.*—Mrs. Spring writes that in her experience of many years she has never come across a baby that would go all night without food. She always feeds an infant about every three hours during the night. Mrs. Spring says "comforters" are shameful things, and we quite agree with her.

(43) Any *nurse* who stood by and saw quietly such a dirty trick as treating a burn with grated unwashed potatoes would be unworthy of her title. A nurse ought to know the proper dressing of a burn.

(44) *Macintosh.*—Can be had from P. B. Gow and Co., 46, Cheapside, E.C.; or from the Argyle Rubber Company, Glasgow, price 2s. 6d. per yard, 36 inches wide; or from the Longford Wire, Iron, and Steel Company, Warrington.

(44) *Macintosh.*—At any large drapery establishment, I should say, in any town of any importance. I know I used to have difficulty in getting the right kind even at a rubber manufactory. Now I can get it at Messrs. Rowntree's Drapery and Furnishing Establishment, Scarborough, where they keep several kinds. The best certainly for a sick bed is the drab, macintoshed both sides, that with the rubber only on one side the material being naturally much less waterproof, and therefore stains easily, and as it is most difficult to remove the stains looks objectionable and uncleanly directly. The kind first mentioned is about four shillings per yard a little over a yard wide. I once got some very good macintosh at a coach builder's before I knew where to get the strong drab kind; it was a dark colour but very strong and serviceable and cheap; it was such as is used for lining horse rugs and carriage aprons, but answered my purpose (district work) admirably.

(45).—Can water and air beds be mended? No, certainly not, you may send them to mend and they will come back with patches on, but they are not one bit better, it is only a waste of money. I have had sixteen years' experience and have tried mending several times always with the same result.—*Sister, Scarborough.*

(45).—I never can get air and water pillows mended, and I have tried many places.—*Warrington.* Water pillows, unless very old, can be repaired at any rubber shop; air pillows cannot be repaired.—*Doris.*

*July.*—(1) Domville's "Manual," price 2s. 6d., published by Churchill (2) The terms have come to be synonymous, though "infirmary" is generally used for wards attached to a union, and "hospital" for those which are open to others except paupers. (3) Of course you must carbolise sheets off a typhoid bed. How can you call yourself nurse and yet have to ask such a question?

*A Reader and Others.*—We pay no attention to anonymous contributions.

*Nurse Bertie.*—Get "Elementary Bandaging and Surgical Dressing," by Pye. Price 2s. (Wright, Bristol.)

*W. B. H.*—We have no room for the lines sent. Your article is in type and will appear shortly.

*Shirley.*—Apply to Colonel John Robertson, C.I.E., 65, Kensington Gardens Square, W. You must train in England first; the scheme is open to all nurses.

*Inquirer.*—You will find a list of Children's Hospitals in the "Hospital Annual," price 2s. 6d., to be had from this office. We have no room here to give such a list.

*Sister Dora.*—At Guy's the lady pupils are specially trained to become sisters; but, as a rule, in the London hospitals a sister is chosen either from the ranks of the paying or the paid probationers wherever a suitable woman for the post can be obtained. This is, of course, by far the best and most just way.

*M. D.*—Apply to the Brassey Holiday Home, St. Leonards-on-Sea, or to Netley Cottage, Claremont Road, Folkestone, or to the Convalescent Hospital, Blackrock, Brighton.

*Nurse.*—We know of no hospital in Gloucestershire which only binds its probationers for one year; the one year's course is, as a rule, only for paying probationers, but you might apply to Whitechapel Infirmary (no salary); or to Clayton Hospital, Wakefield; or Douglas Hospital, Isle of Man.

*To Our Correspondents.*—We are much obliged for the many answers sent to queries in response to our appeal, and hope they may continue to be as numerous. Last week we had unfortunately to omit both letters and queries, owing to a pressure on our space; but this is not likely to occur again.

*Presentation of Screen.*—We have received two letters from members of the First Thousand, asking if the screen presented to Mr. Burdett could not be photographed. We understand this can be done, and will be carried out by Messrs. Elliott and Fry; then if any subscribers to the screen like to have copies they can obtain them direct from the photographers, 55, Baker Street, Portman Square, W.

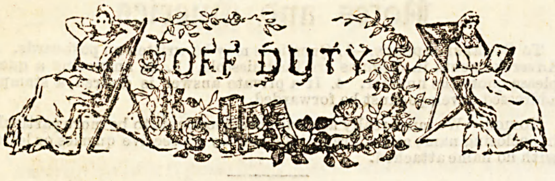

### The Celibates of the Future.

It is well sometimes in our "off duty" time to wander a wee bit away from the subject of wards and find out what our sisters in other professions are thinking about. Doubtless, this was the reason which took several nurses to the Somerville Club for Ladies last week, when Dr. Philpot discoursed on "The Celibates of the Future," Miss Foley, M.A. in the chair.

The subject was approached from the medical standpoint, yet Dr. Philpot wandered far from scientific possibilities into regions of fiction, such as the soul of Walter Besant loveth. We are all to live for posterity, according to Dr. Philpot, and to secure this the weakly and diseased are to retire into monasteries, while the strong and healthy remain in the world and marry. Great was the scorn the lecturer poured on sanitarians, with their filters and drain-traps and remedial measures; the only way, according to Dr. Philpot, to raise the standard of health was to breed healthy children; in other words, to attend to the birth-rate, and let the death-rate take care of itself. The French when their vines were attacked by phylloxera instead of trying to kill the pest planted American vines which were so strong they could withstand the disease. So in order to strengthen the human stock against disease the weakly are to retire into monasteries and give to the world the fruit of their intellect alone. The lecturer was a bold young man, and though he was addressing an audience of women, he spoke far more directly than we care to do, and his example was followed by the critics, who joined in the subsequent debate. One could not help wondering what our grandmothers would have thought if they had been present at the Somerville lecture last week. However, the motto of to-day is realism at all costs—in our books, our plays, our lectures, the plain unvarnished truth of even the darkest side of life. The members of the Somerville have mostly hard heads and short hair; they listened quietly to the wild scheme described to them, then they got up and "heckled" the lecturer with a vengeance. Who was going to decide who should retire to the new monasteries? Were the inmates to be sent in by force? Would such a scheme appeal to the millions, or only to the cream of humanity? and supposing it applied to the cream only, the millions would go on handing down disease, while the cream of our intellects would get no chance with heredity? In fact, "monastery" was evidently only a new name for lunatic asylum, and none of the healthy idiots outside would pay the least attention to the intellectual fruits of the sickly geniuses within its walls. Besides, though the modern abhorrence of marriage is very strong in the ranks of the Somerville members, they were not ready to accept Dr. Philpot's second contention—that it is the duty of the healthy to marry. The debate was short, and to the point; the wild scheme for the celibates of the future had not a leg to stand on when the Somerville members had done with it. With a sigh the lecturer rose, and humbly thanked the ladies for the pleasant half-hour they had given him—a remark which roused some laughter.

Of course the great truth that we do owe a duty to posterity, and that those who hand down to their children hereditary disease are cruel, if not criminal, was never denied. But surely the best way to secure this is to rouse public opinion and train the mind, not chain the body. A little freedom must be left to the poor creatures of the future. The thought of matrimony would be simply fostered because, when forbidden, the unfortunate victim was locked up to think over his loss; but give him work and interest and a brilliant existence as a soldier or a sailor, say, and he will be more likely to fulfil his destiny and his duty. Let Dr. Philpot preach celibacy far and wide if he will, and especially make known the evils of hereditary disease, but let him not advocate any making of communities with impossible rules and unnatural lives. We have had enough of the morality of the monasteries, of the religion of the cell and the scourge. Work worth the doing is to be found by everyone who enters the service of humanity, and plenty of trials to tame the spirit and teach forbearance. There are some devils we know which go not out by fasting and prayer, but only by labour and forgetfulness of self.

### Where to Go.

Dr. E. Symes Thompson will deliver the Gresham Lectures on Physic at Gresham College, Basinghall Street, at six p.m. on April 7th, 8th, 9th, and 10th; admission free.—At the Royal Institution, Albemarle Street, Edward E. Klein, M.D., F.R.S., will deliver three lectures on "Bacteria: their Nature and Functions" (the Tyndall lectures), on Tuesdays, April 28th, May 5th and 12th, at three p.m.; half a guinea. —A course of six lectures on "The Origin and Growth of the Idea of God as illustrated by the Comparative History of Religions," will be delivered in French by Count Goblet D'Alviella, Professor of History of Religions in Brussels University, at the Portman Rooms, Baker Street, on April 15th, 16th, 20th, 21st, 27th, and 28th, at five p.m. Admission to the course of lectures will be by ticket, without payment. Persons desirous of attending the lectures are requested to send their names and addresses to Messrs. Williams and Norgate, 14, Henrietta Street, Covent Garden, W.C., not later than April 11th.—At Kew Gardens great patches of crocuses are in blossom, and the orchid house is at its best. Kew Gardens can be reached from any of the Underground stations, the return fare being from one to two shillings. The Gardens are open free daily (Sundays included), after one p.m.

### Amusements and Relaxation.

### SPECIAL NOTICE TO CORRESPONDENTS.

**Second Quarterly Word Competition commences April 4th, ends June 27th, 1891.**

Competitors can enter for *all* quarterly competitions, but no competitor can take more than *one first prize* or *two* prizes of any kind during the year.

Proper names, abbreviations, foreign words, words of less than four letters, and repetitions are barred; plurals, and past and present participles of verbs, are allowed. Nuttall's *Standard* dictionary only to be used.

N.B.—Word dissections must be sent in WEEKLY not later than the first post on Thursday to the *Prize Editor*, 140, Strand, W.C., arranged alphabetically, with correct total affixed.

The word for dissection for this, the FIRST week of the quarter, being "GRASSE."

| Names. | March 26th. | Totals. | Names. | March 26th. | Totals. |
|---|---|---|---|---|---|
| Reynard | — | 77 | Woodbine | — | 25 |
| Reldas | 30 | 606 | Madame B. | — | 25 |
| Tinie | — | 30 | Shakespeare | — | 59 |
| Patience | — | 76 | Smyrna | — | 259 |
| Jenny Wren | 25 | 531 | Southwood | — | 102 |
| Agamemnon | 29 | 596 | Gipsy Queen | — | 21 |
| Wyameris | — | 391 | Snowball | — | 19 |
| E. O. | 26 | 587 | Rita | — | 88 |
| Ecila | — | 283 | Mortal | — | 16 |
| Hope | 30 | 601 | Nurse Annie | — | 15 |
| M. W. | 26 | 583 | Carmen | — | 11 |
| Qu'appelle | 30 | 583 | Grannie | — | 45 |
| Nil Desperandum | 30 | 595 | Amie | — | 30 |
| Lady Betty | 30 | 552 | M. R. | — | 25 |
| H. A. S. | — | — | Primrose | — | 24 |
| Sister Jack | — | 62 | Nurse J. S. | 25 | 349 |
| Crystal | — | 203 | B. A. C. | — | 223 |
| | | | Theta | 30 | 148 |

**Notice to Correspondents.**

N.B.—Each paper must be signed by the author with his or her real name and address. A *nom de plume* may be added if the writer does not desire to be referred to by us by his real name. In the case of all prize-winners, however, the real name and address will be published.

